# Influence of environmental dust in transformation capacity of S*treptococcus pneumoniae*

**DOI:** 10.1590/1678-4685-GMB-2020-0028

**Published:** 2020-12-11

**Authors:** José Diogo de Oliveira, Carlos Fernando Macedo, João Paulo de Oliveira Guarnieri, Thaís Holtz Theizen, Daisy Machado, Marcelo Lancellotti

**Affiliations:** 1 Universidade Estadual de Campinas, Instituto de Biologia, Departamento de Bioquímica e Biologia Tecidual, Campinas, SP, Brazil.; 2 Universidade Estadual de Campinas, Faculdade de Ciências Farmacêuticas, Campinas, SP, Brazil.

**Keywords:** Streptococcus pneumoniae, transformation, environmental pollutants, quorum sensing, dust

## Abstract

*S. pneumoniae*, commonly known as pneumococcus, is a naturally competent Gram-positive bacterium and is the major cause of pneumonia in elderly and children in developing countries. This pathogen is associated with respiratory diseases affected by pollution. The objective of this work was determining the effect of ash and environmental dust from the burning of sugarcane on pneumococci bacterial transformation. The transformation capacity of the Pn360 pneumococci strain was performed using the assays of DNA donor of mutant for *luxS* gene. Thus, the transformation tests were performed in contact with dust collected in the southwestern region of Brazil (important region where burning of sugar cane is present in the agriculture). The use of degradative practices in the sugar cane agriculture in Brazil was involved in the transformation capacity of the *S. pneumoniae*. This phenomenon includes important consequences for public health concerning to resistance acquisition and new virulence factors of this important infection. In conclusion, we obtained important results concerning the action of environmental pollution in *Streptococcus pneumoniae* transformation, increasing the DNA acquisition for this pathogen.

## Introduction

The relationship between high rates of exposure to the smoke from the burning of biomass with the incidence of respiratory diseases was described by several authors in several places around the world (Arbex *et al.* 2007). Studies performed in the state of Louisiana showed that months with the highest incidence of asthma in the city hospital of Houma were october, november and december, coinciding with the months of burning of sugarcane in the region (Boopathy *et al.* 2002). As previously reviewed in the literature, respiratory diseases are intricately linked to the burning of sugarcane (Arbex *et al.* 2007). However, the studies done so far have placed the incidence of other diseases of the respiratory tract with great social impact, as in the case of pneumonia and meningitis. Among the pathogens causing such diseases, *Streptococcus pneumoniae* stands out (Joloba *et al.* 2001; Joloba *et al.* 2010). *S. pneumoniae,* commonly known as pneumococcus, is a naturally competent gram-negative bacterium and is the major cause of pneumonia in elderly and accounts for about 5 million deaths of children per year in developing countries (Asturias *et al.* 2003; Duke 2005; Zaidi *et al.* 2009). In these countries, the rate of pneumococcal infections in children up to six years of age is three to eight times higher than in Western European and North American countries (Hausdorff *et al.* 2001). Estimates indicate that about two children die per hour in Latin America due to pneumococcal diseases (Ochoa *et al.* 2010). As previously reviewed in the literature, respiratory diseases are linked to the burning of sugarcane. However, the studies done so far have placed the incidence of such diseases attributed only to pathophysiological and histopathological factors of such events. For instance, one study has shown that the mesoporous silica nanoparticle has a potentiating effect on *S. pneumoniae* transformation. When the transformation process was carried out in the presence of the silica nanoparticle (SBA 15 and SBA 16), a larger number of transforming bacteria were observed when contrasted with the control, in which the transformation was performed without nanoparticles. The objective of this work was to observe the effect of ash from the burning of sugarcane on bacterial transformation, since this compound is rich in particulate matter. Our tests were based in the mimicking of lung environment with the use of A549 cells to verify the effect in transformation pneumococci capacity.

## Material and Methods

### Bacterial Growth

The strain of *Streptococcus pneumoniae* used in this study was PN360 ATCC33400. The bacteria were cultured in an incubator at 37 ºC and 5% CO_2_ in BHI medium containing 5% horse blood. The ashes were obtained by two forms: combustion of dried sugarcane and collection of environmental ashes in region near the city of Campinas. Both dissolved in water and then sterilized by autoclaving. The DNA used in this work was obtained from a mutant strain of *S. pneumoniae* (Sp360Δ*luxS*) as described by Amstalden *et al*. (2019).

### Cell cultures

The cell cultures were cultivated as described by Hollanda *et al.* (2014) and Varela *et al.* (2014). Cell lines A549 (pulmonary epithelial carcinoma) were obtained at the Institute Adolfo Lutz, Brazil. They were cultivated at RPMI medium 1640 (Cultilab, Campinas, Brazil) with 10-20% Bovine Fetal Serum (BFS) and maintained at 37° C with 5% CO_2_. After the semiconfluent cell layer formation, the cells were trypsinized with solution at 2.5 g/L EDTA 0.2 g/L (Cultilab, Campinas, Brazil) and transferred to a 24-well polystyrene plate. Each well received 1 mL of cell culture and the cell concentration after its trypsinization was 1x10^6^ cells/mL. 

### Transformation

The DNA recombination protocol for plasmid cloning, PCR reaction, insertion of the resistance cassette and transformation were described previously by Varela *et al.* (2014), replacing the nanoparticles by ashes in the cited articles. Mutants obtained by homologous recombination were verified by PCR following the protocol described in Amstalden *et al.* (2019). Briefly, the Sp360 bacterial strain was grown to a final concentration of 10^8^ cfu/mL in a final volume of 5 mL. The bacterial solution was diluted 1:100 resulting in a final volume of 10 mL in BHI medium in which CSP-1 (synthesized by Aminotech, Brazil) was added at a final concentration of 150 ng/mL. After this period, 1 µg of genomic DNA (diluted in 10 µL of water) from the SP360 Δ*luxS* strain was added along with 200 μg of ashes, resulting in a final volume of 1 mL. The negative control was done without the presence of ashes. Bacteria were incubated for 24 hours at 37 ºC and 5% CO_2_ in 24-well plates for 6 hours. The transformation efficiency was measured by transferring 1 mL of each replicate (with addition of control and ash) to chocolate agar plates with the addition of 40 mg/mL spectinomycin and then incubated at 37 ºC for 24 hours. The transformed CFUs were then counted and analyzed through PCR reaction to check for the presence of the gene used (Δ*luxS*: : Ω*aaDA*).

### Statistical analysis

Analysis were made by comparing the transforming colonies treated with ashes and dust, with the CFUs formed without the presence of such compounds. Statistics tests were performed by ANOVA test of significant results.

## Results

**Figure 1 -:**
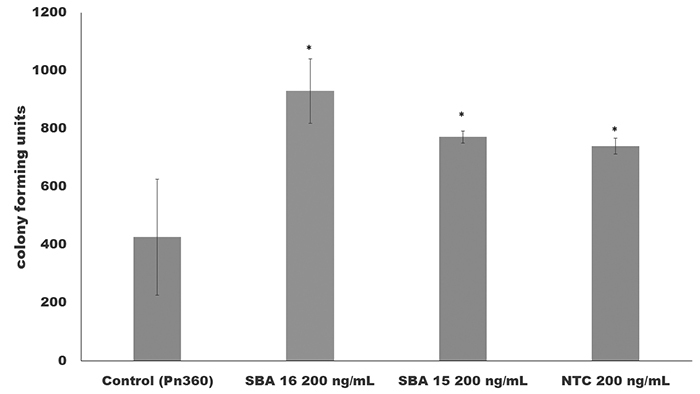
Effect of the synthetic nanoparticles SBa 15, SBa16 and multiwalled carbon nanotubes (NTC) in pneumococci transformation. In “x” the conditions of transformation (control not exposed at nanoparticles structures and mesoporous silica SBa15, 16 and multiwalled carbon nanotubes) and axe “Y” the colonies forming units obtained from transformations process in blood agar supplemented with spectinopmycin. The concetration of those nanostructures were equally distributed in well triplicate tests at 200 ng/mL. *All the nanostructures showed significant results when compared with control preparation (p values <0.05).

The transformation experiments using the *S. pneumoniae* strain ATCC33400 was performed using as donor DNA the mutant for quorum sensing gene *luxS*. The transforming units obtained from the mix of the peptide, bacterium and synthetic carbon nanotubes (Mattos *et al*., 2011), and mesoporous silica SBa15 and SBa16. The tests were performed in triplicate and the results shown in the Figure 1. These results indicate an increase in the number of the transforming units’ colonies in the tests in presence of SBa15, SBa16 and multiwalled carbon nanotubes (MWCNT). The significant results were discriminated in the legend of the Figure 1.

**Figure 2 -:**
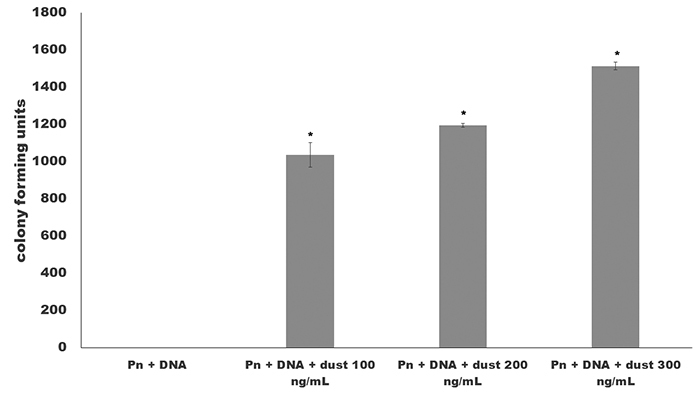
Effect of different ashes concentration in pneumococci transformation. The *S. pneumoniae* PN360 ATCC33400 was exposed to 100, 200 and 300 ng/mL of ashes obtained from burnt sugar cane. The results showed the colony forming units obtained in triplicate tests performed similarly to protocols using nanoparticles. The increases of the colony forming units obtained from ashes use were superior than compared with control test. *All the values showed significant results when compared with control sample (p values <0.05).

In our analysis, we collected the environmental dust in the Americana city in southwest region in Brazil (22°44’21’’S , 47°19’53’’W) and submitted it to the same test in pneumococci transformation. Figure 2 showed the effect of this dust collected in the period of May to July of 2014. The results showed an increase of the pneumococci transformation in comparison with the control without environmental dust (zero colony forming units). The ashes had no effect on bacterial growth (data not shown).

**Figure 3 -:**
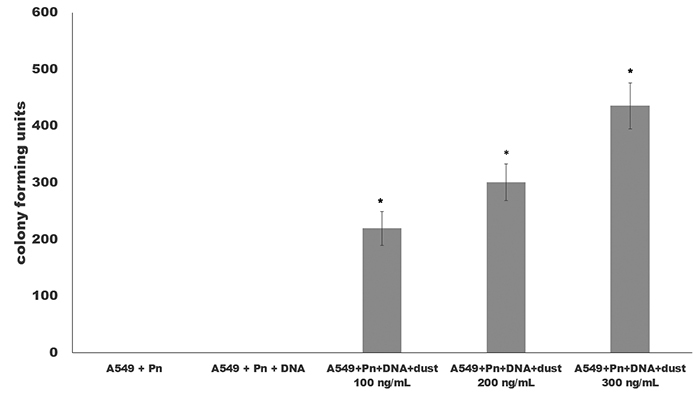
The use of a system mimicking the respiratory tract by A549 cells use (adenocarcinoma from lung) and its action in pneumococci transformation. The colony forming units obtained from the test where the pneumococci cells were exposed to A549 cells microenvironment and environmental dust in a Brazilian region where the burning of sugar cane is a practice in the agricultural management. All concentrations of dust (100, 200 and 300 ng/mL) were capable to increase the transformation capacity of pneumococci cells in contact with A549 microenvironment. *All the values showed significant results when compared with control sample (p values <0.05).

Also, for mimicking the human upper tract and its environment, the transformation process occurred over a cellular monolayer of a lung lineage cell - the A549. After the exposition to the particulate material, Figure 3 showed the transforming colony units obtained in these tests. The data demonstrated the significant increase from zero transforming units to more than 1000 colony-forming units per milliliters.

## Discussion

The role of nanoparticles in the transformation process of natural competent bacteria is described by our group in several works using purified nanostructures, as the multiwalled carbon nanotubes and the mesoporous silica with action in *Neisseria meningitidis* and *Haemophilus influenzae*. Recently, we described the use of mesoporous silica in pneumococci transformation also showing the involvement of this pathogen in acquiring virulence genes and resistance characteristics by transformation. The action of these nanoparticles in natural environment is our aim in the transformations studies of this bacterial pathogenesis (Amstalden *et al.*, 2019). 

This work showed evidences of the environmental action of nanoparticles obtained from the burning of sugar cane and its consequences in public health. In the experiments involving nanoparticles, the same results were observed with an increase of the transformation capacity in *N. meningitidis* and *H. influenzae* (Mattos *et al.*, 2011; Hollanda *et al.*, 2014). These results were confirmed using the dust collected from the region where our study was performed together with a lung mimicking system with A549 cells line. The results described in Figure 3 showed the effect of nanostructures present in environmental dust.

Furthermore, this study reinforced the protective effect played by the dust in pneumococci DNA during events of bacterial transformation. This is the first data using the environmental dust in a *S. pneumoniae* transformation observing the effective action in this phenomenon. In conclusion, this work obtained important results concerning the action of environmental pollution in *Streptococcus pneumoniae* transformation, increasing the DNA acquisition for this pathogen. The characteristic of this process linked with the protective capacity of the dust (and their nanoparticulated material) against DNase action promotes an increase in the transformation capacity of this bacterium. Also, this pneumococci capacity increases new cases of infection caused by this bacterium and might be associated with antibiotic resistance acquisition.
